# Compositional Analysis of Longshan Period Pottery and Ceramic Raw Materials in the Yongcheng Region, Henan Province

**DOI:** 10.3390/ma18122681

**Published:** 2025-06-06

**Authors:** Linyu Xia, Yinhong Li, Ge Zhang, Jialing Li, Li Jaang

**Affiliations:** 1 School of Archaeology and Cultural Heritage, Zhengzhou University, Zhengzhou 450001, China; xialinyuzk@163.com (L.X.); li06082024@163.com (Y.L.); lijaangchina@gmail.com (L.J.); 2 Historical and Cultural Heritage Protection Research Center, Zhengzhou University, Zhengzhou 450001, China; 3 Yellow River Institute of Hydraulic Research, Yellow River Water Conservancy Commission, Zhengzhou 450003, China; 4 Key Laboratory of Lower Yellow River Channel and Estuary Regulation, Ministry of Water Resources, Zhengzhou 450003, China; 5 Yellow River Laboratory, Zhengzhou 450003, China; 6 School of Water Conservancy and Transportation, Zhengzhou University, Zhengzhou 450001, China; jialingli1120@163.com

**Keywords:** Yongcheng region, Longshan culture, pottery composition analysis, ceramic manufacturing techniques, raw material sources

## Abstract

This study systematically analyzes the composition and microstructure of Neolithic pottery unearthed from the Dazhuzhuang, Likou, and Biting Sites in the Yongcheng District using techniques such as X-ray fluorescence spectroscopy (XRF), X-ray diffraction (XRD), infrared spectroscopy (IR), and scanning electron microscopy with energy-dispersive spectroscopy (SEM-EDS). The results show that although the raw materials for pottery at the three sites were likely sourced from nearby ancient soil layers, significant differences in chemical composition and manufacturing techniques are evident. Pottery from the Dazhuzhuang Site is mainly composed of argillaceous gray pottery, with relatively loose raw material selection and a wide fluctuation in SiO_2_ content (64.98–71.07%), reflecting diversity in raw material sources. At the Likou Site, argillaceous black pottery predominates, characterized by higher Al_2_O_3_ content (17.78%) and significant fluctuations in CaO content (1.46–2.22%), suggesting the addition of calcareous fluxes and the adoption of standardized manufacturing techniques. Pottery from the Biting Site mainly consists of argillaceous gray pottery, showing higher Al_2_O_3_ content (17.36%), stable SiO_2_ content (65.19–69.01%), and the lowest CaO content (0.84–1.81%). The microstructural analysis further reveals that the black pottery (from the Likou Site) displays dense vitrified regions and localized iron enrichment. In contrast, the gray pottery (from the Dazhuzhuang and Biting Sites) shows clay platelet structures and vessel-type-specific differences in porosity. This research provides important scientific evidence for understanding raw material selection, manufacturing techniques, and regional cultural interactions in the Yongcheng area during the Longshan Culture period.

## 1. Introduction

The pottery manufacturing techniques during the Neolithic period in China had already reached a high level of maturity, with diverse vessel forms and strong functional designs. As a representative culture of the Late Neolithic period in China, the Longshan Culture played a key role in developing both cultural evolution and pottery technology. The Longshan Culture in the Yongcheng District, as an important and distinctive regional type within the Longshan cultural system, has attracted significant attention from scholars both in China and abroad. In 1936, Li Jingdan conducted archeological surveys in the Shangqiu and Yongcheng areas in search of the origins of the Shang civilization. He discovered several Longshan Culture remains along both sides of the Huai River in Yongcheng and carried out small-scale excavations at the HeiguDui Site, the Zaolütai Site, and the Caoqiao Site, uncovering a collection of pottery, stone tools, and bone artifacts [1]. In the late 1970s, to explore the social conditions in eastern Henan at the end of the primitive society, the Institute of Archaeology of the Chinese Academy of Social Sciences and the Shangqiu Cultural Relics Administration conducted three rounds of surveys in the counties of Shangqiu, discovering 17 Longshan Culture sites [2] and carrying out excavations at the Wangyoufang Site [3] and the Heigudui Site [4] in Yongcheng. The Wangyoufang Site is considered the most representative, with thick cultural deposits and rich artifact types, and the Longshan Culture remains from this site are referred to as the Wangyoufang type. Subsequently, further discoveries of Longshan Culture remains have been made in eastern Henan, including at the Duanzhai Site in Dancheng, the Pingliangtai Site in Huaiyang, and the Luantai Site in Luyi, as well as the Qingliangshan Site in Xiayi and the Lutai Gang Site in Qixian, Kaifeng. These sites have all yielded Longshan Culture remains and are generally classified under the Wangyoufang type. In 1995, a Sino-American joint archeological team excavated the Shantaisi Site in Zhecheng, discovering a large number of Longshan cultural features and artifacts, such as large rammed-earth platforms and sacrificial cattle pits, confirming its central position in the Longshan Culture of eastern Henan [5]. In 2002, the Department of Archaeology at Zhengzhou University surveyed 24 ancient cultural sites in Shangqiu, including four Longshan Culture sites in Yongcheng: the Hongfu Site, the Zhaozhuang Site, the Mingyangsi Site, and the Zaolütai Site [6]. In 2022, to further clarify the distribution of cultural relics and settlement hierarchy of the Longshan Culture in the Huai River basin of Yongcheng, the Department of Archaeology at Zhengzhou University carried out a comprehensive systematic survey and exploration of the cultural remains in the region.

With the continuous publication of archeological excavation data from Longshan Culture sites, the study of Longshan Culture has produced abundant results. Traditional archeological research has primarily focused on the cultural chronology and origins, especially in classifying and forming regional types, such as the Wangyoufang type [7] and the Wangwan type [8] of the Longshan Culture. Secondly, studies have addressed the geographical distribution of the culture. Scholars such as Liang Sicheng [9], An Zhimin [10], and Yang Zifan [11] have proposed different views on the extent of the Longshan Culture, with three prevailing opinions regarding the western boundary of the Haidai Longshan Culture distribution area [12]. Thirdly, many specialized studies have been carried out on the nature and function of sites and have unearthed artifacts and features—especially tombs and typical pottery vessels. By conducting typological research on representative pottery, scholars have explored issues related to their distribution, manufacturing techniques, morphological evolution, functions, and social significance.

With the adoption of experimental archeological methods, Chinese researchers have increasingly employed techniques such as X-ray fluorescence spectroscopy (XRF), wavelength-dispersive X-ray fluorescence (WDXRF), and scanning electron microscopy–energy dispersive spectroscopy (SEM-EDS) to analyze the chemical composition and firing techniques of Longshan Culture pottery. These methods have facilitated deeper investigations into raw material sources, production processes, and technical strategies. For example, Li Minsheng and Huang Suying conducted chemical and physical analyses on pottery sherds from different periods at the Taosi Site, shedding light on ceramic manufacturing techniques during the Miaodigou Phase II and Longshan periods [13]. Chen Qianqian and Yang Yuzhang used WDXRF to study Neolithic pottery from the Jiahu Site in Henan and the Xiaohuangshan Site in Zhejiang [14]; Gao Shoulei applied XRF to analyze the composition of pottery from the Hongshan Culture at the Niuheliang Site [15]. As pottery is one of prehistoric cultures’ most important material remains, its manufacturing technology and social function have become core issues in international archeological research [16,17,18,19,20]. Internationally, scholars widely use techniques such as XRF, X-ray diffraction (XRD), SEM-EDS, and Raman spectroscopy to analyze the composition, mineral structure, and microstructure of pottery, revealing the technological development of ceramic production [21,22,23,24]. For example, Maja Gajić-Kvašćev used EDXRF to study the composition of pottery from the Velika Humska Čuka Site in Serbia, providing evidence of cultural exchange influences [25]. Laura Teodorescu and colleagues combined XRD and SEM-EDS to analyze the raw materials and production techniques of Dacian pottery [26]. Philippe Colomban and others applied XRF and Raman spectroscopy to detect different chemical compositions in ceramics, proving that as early as the 12th century CE, Islamic potters had mastered highly advanced ceramic techniques [27]. These international research paradigms have provided important references for the scientific and systematic development of ceramic studies in China [28,29,30,31,32].

However, current scientific archeological research still faces challenges, such as limited analytical methods and lacking a comprehensive technical system that integrates multiple techniques and interdisciplinary approaches. This has hindered a full understanding of the complexity of pottery manufacturing techniques and the diversity of raw material sources. Based on this research context, the present study adopts an integrated approach combining traditional archeology and materials science. By applying both typological and scientific methods and using a combination of analytical techniques, including XRF [33,34,35,36], XRD, infrared spectroscopy (IR), and SEM-EDS, we aim to carry out systematic analysis at the levels of chemical composition, mineral structure, and microscopic morphology [37,38,39,40,41]. This multi-technical approach overcomes the limitations of relying on single methods, providing more reliable data for identifying pottery composition and raw material sources.

**Figure 1:**
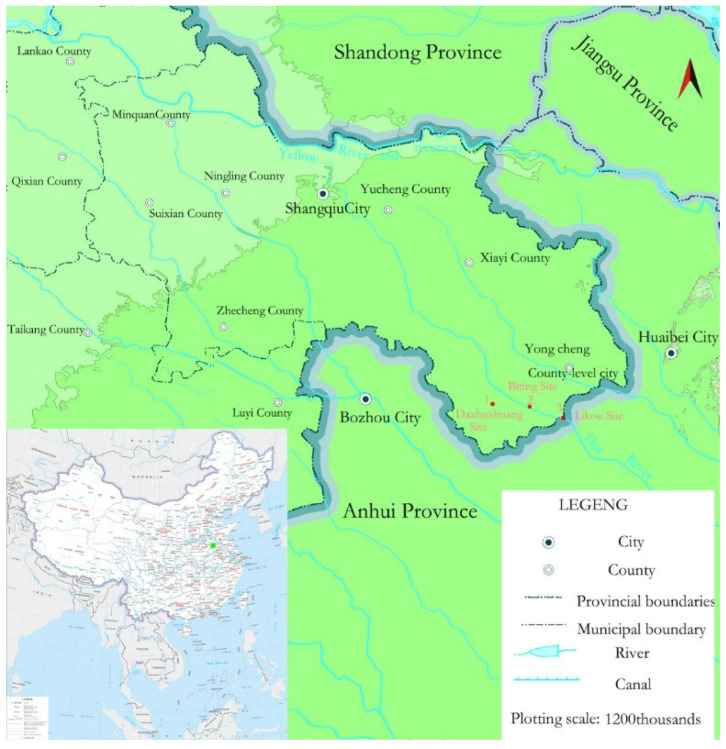
Schematic map of site distribution locations.

In 2022, the Department of Archaeology at Zhengzhou University conducted a systematic survey and exploration in the Huai River basin of the Yongcheng District, identifying 17 archeological sites containing cultural remains from the Longshan Culture, as shown in Figure 1. Among them, the Dazhuzhuang Site (16,800 m^2^), the Biting Site (40,000 m^2^), and the Likou Site (90,000 m^2^) are located at the junction of Henan, Anhui, and Shandong Provinces. These sites serve as a crucial area for interaction between the Wangyoufang type of the Longshan Culture in eastern Henan and adjacent cultural regions. With well-defined hierarchical settlement structures, these sites yielded abundant pottery sherds from the Longshan period, featuring diverse types and representative vessel forms. For this reason, pottery from these three sites was selected as the focus of the present study. This research integrates domestic and international advances in pottery studies through the systematic sampling and analysis of typical pottery sherds unearthed from the Dazhuzhuang, Biting, and Likou Sites [42,43,44,45,46]. It applies a multi-technical and interdisciplinary approach to investigate Longshan Culture pottery in the Yongcheng District comprehensively. The objective is to clarify the raw material sources and technological characteristics of these ceramics during the Longshan period, thereby uncovering the intrinsic relationship between ceramic manufacturing and regional cultural interaction. This study aims to provide new empirical evidence for understanding regional variation within the Longshan Culture and the pathways of ceramic technological transmission.

The specimens analyzed from the three sites are introduced as follows:

1. Dazhuzhuang Site

The Dazhuzhuang Site is on the northwest side of Dazhuzhuang Village, Peiqiao Town, Yongcheng District. It is approximately 120 m wide from north to south and 140 m long from east to west, covering an area of about 16,800 square meters. The cultural deposits are 0.8–2.1 m thick. Collected artifacts are mainly pottery sherds, with a few shells and animal bone remains. The pottery primarily dates back to the Longshan culture and Han periods. Pottery from the Longshan Culture is mainly argillaceous gray pottery, followed by argillaceous brown pottery and argillaceous black pottery. Decorations are primarily cord marks and plain surfaces, followed by basket patterns, along with a few string patterns and grid patterns. Vessel types include storage jars (ceramic containers for liquids or food), the Yan (a cooking vessel functionally similar to a modern steamer, comprising an upper zeng for holding food and a lower li for water, separated by a perforated grate to allow for steam circulation), the Ding (tripod or quadripod cooking vessels with handles, typically round with three legs and two ears, though rectangular versions exist), the Dou (food-serving vessels with tall ring feet), urns (storage vessels with a narrow mouth and bulging belly), bowls (round concave dishes for food), and cups.

A total of 15 specimens were selected for testing, including vessel types such as storage jars, basins (wide-mouthed, narrow-based containers for liquids, food, or washing), cups (liquid-holding vessels), Ding legs, Yan legs, and handles (lateral protrusions for grasping). The majority are argillaceous gray pottery, with a few argillaceous brown pottery pieces. Decorations are mainly grid patterns and plain surfaces.

**Figure 2:**
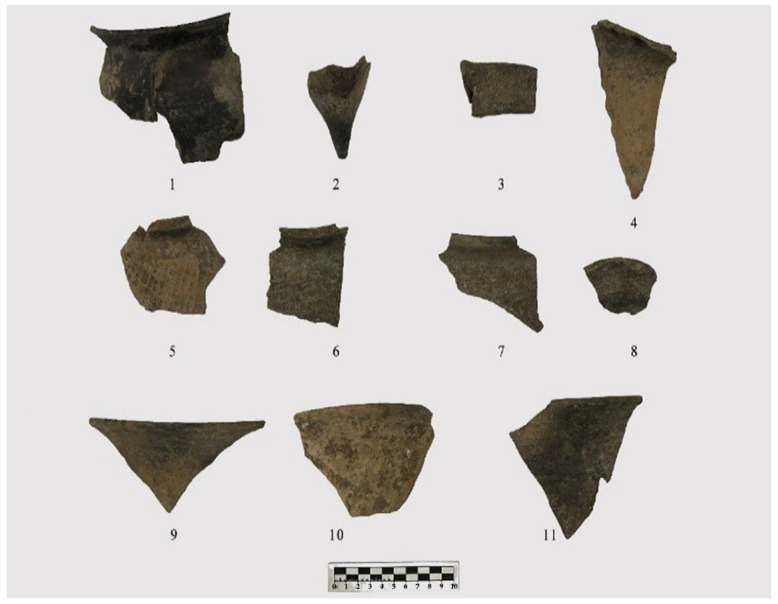
Pottery specimens from the Longshan Culture period at the Dazhuzhuang Site. (**1**) Storage jar (2023YPDH1:1), (**2**) Yan leg (2023YPDH1:23), (**3**) handle (2023YPDH1:17), (**4**) Ding leg (2023YPDH1:21), (**5**) storage jar (2023YPDH1:9), (**6**) storage jar (2023YPDH1:4), (**7**) storage jar (2023YPDH1:5), (**8**) cup (2023YPDH1:19), (**9**) basin (2023YPDH1:13), (**10**) basin (2023YPDH1:12), and (**11**) Basin (2023YPDH1:11).

**Figure 3:**
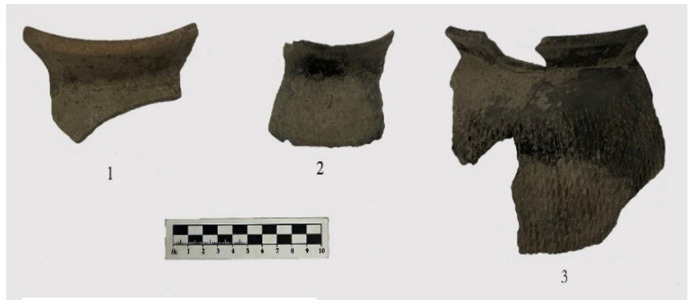
Pottery specimens from the Longshan Culture period at the Dazhuzhuang Site. (**1**) Storage jar (2023YPDH1:16), (**2**) basin (2023YPDH2:12), and (**3**) storage jar (2023YPDH1:5).

Seven storage jars were examined, including six argillaceous gray pottery specimens and one argillaceous brown pottery specimen. The six argillaceous gray pottery pieces exhibit flared rims, folded rims, square lips, contracted necks, and sloping shoulders. Specimen 2023YPDH1:1 features a globular body and is preserved below the belly. The surface is plain (Figure 2(1)). Specimen 2023YPDH1:4 is preserved below the shoulder, which is decorated with grid patterns (Figure 2(6)). Specimen 2023YPDH1:5 has a round lip and is preserved below the shoulder. The shoulder is decorated with diamond-shaped grid patterns (Figure 2(7)). Specimen 2023YPDH1:16 has a square lip with a circumferential groove on the lip surface and is preserved below the shoulder. The shoulder is decorated with grid patterns (Figure 3(1)). Specimen 2023YPDH2:1 has a grid pattern decoration on the shoulder. Specimen 2023YPDH2:5 features a round lip and a curved body, with the lower belly preserved. The exterior is decorated with medium-sized cord marks (Figure 3(3)).

Specimen 2023YPDH1:9 is an argillaceous brown pottery storage jar. It features a flared rim, a folded rim, a square lip with a circumferential groove on the inner edge, a contracted neck, sloping shoulders, and a curved body. The portion below the belly is preserved. The exterior has diamond-shaped grid patterns (Figure 2(5)).

Four basins were examined, including three made of argillaceous gray pottery. These basins have open mouths, round lips, and obliquely straight bodies, with parts preserved below the belly. The surfaces are plain. Specimens include 2023YPDH1:13 (Figure 2(9)), 2023YPDH1:11 (Figure 2(11)), and 2023YPDH2:12.

One specimen is made of argillaceous brown pottery, featuring an open mouth, a round lip, an obliquely straight body, and preservation below the belly. The surface is plain (Specimen 2023YPDH1:12) (Figure 2(10)).

One cup was identified. Specimen 2023YPDH1:19 is an argillaceous gray pottery piece with an open mouth, a square lip, a circumferential groove on the inner edge of the rim, a shallow body, and a flat base. The surface is plain (Figure 2(8)).

One Ding leg was found. Specimen 2023YPDH1:21 is an argillaceous brown pottery piece. It has a side-triangular flat leg with a solid root. There are six depressions on the outer side of the leg root. The surface is plain (Figure 2(4)).

One Yan leg was recovered. Specimen 2023YPDH1:23 is made of argillaceous gray pottery and features a pouch-shaped leg with a tall, tapered, solid root. The surface is plain (Figure 2(2)).

One handle was documented. Specimen 2023YPDH1:17 is an argillaceous gray pottery piece with a bridge-shaped handle. The surface is plain (Figure 2(3)).

2. Biting Site

The Biting Site is located southeast of Bianzhuang Village, Xinqiao Town, Yongcheng District. The central, western, and northern parts of the site are overlain by the town’s grain storage facility. The site is irregular in shape and covers an area of approximately 40,000 square meters. Collected artifacts mainly date back to the Longshan and Shang culture periods. Pottery sherds from the Longshan Culture period are primarily composed of argillaceous gray and argillaceous gray-black pottery, along with some argillaceous polished black pottery and argillaceous brown pottery. There are also small quantities of sand-tempered gray/brown pottery and shell-tempered brown/gray pottery. Decorative patterns are dominated by basket patterns, square patterns, and plain surfaces, followed by cord-marked patterns and a few string patterns. Common vessel types include storage jars, basins, bowls, and Ding.

A total of eight specimens were selected for testing, including storage jars, basins, bowls, and Ding legs. Most are made of argillaceous gray pottery, with decorative patterns mainly featuring square patterns, cord marks, and plain surfaces.

**Figure 4:**
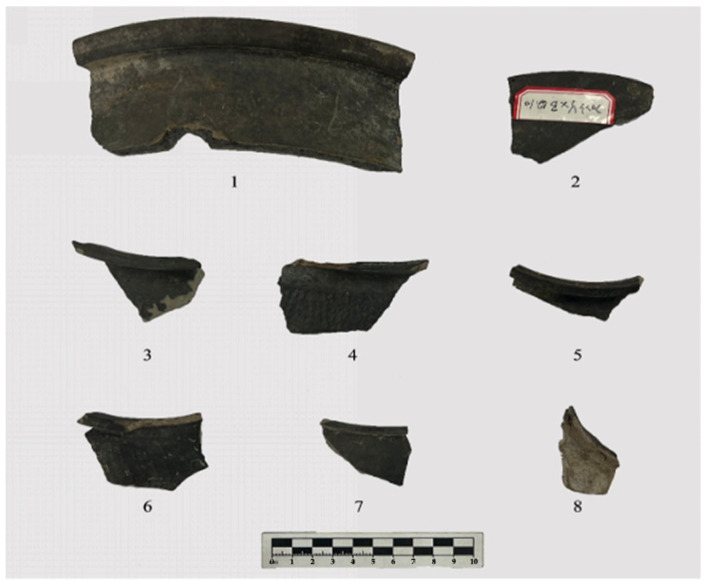
Pottery samples from the Longshan Culture period. (**1**) Basin (2023YXBP⑤:1), (**2**) bowl (2023YXBP⑤:10), (**3**) storage jar (2023YXBP⑤:7), (**4**) storage jar (2023YXBP⑤:21), (**5**) storage jar (2023YXBP⑤:13), (**6**) storage jar (2023YXBP⑤:8), (**7**) storage jar (2023YXBP⑤:14), and (**8**) Ding leg (2023YXBP⑤:19).

Five storage jars, all made of argillaceous gray pottery, have flaring mouths, folded rims, and square lips (Specimen 2023YXBP⑤:7: argillaceous gray pottery). A groove is present inside the lip, and it has a contracted neck and a sloping shoulder but is missing below the shoulder. The shoulder is decorated with a square pattern (Figure 4(3)) (Specimen 2023YXBP⑤:21: argillaceous gray pottery). It has a contracted neck and a sloping shoulder but is missing below the shoulder. The shoulder is decorated with a cord-marked pattern (Figure 4(4)) (Specimen 2023YXBP⑤:13: argillaceous gray pottery). A groove is present inside the lip, and it has a contracted neck, a sloping shoulder, and a plain surface but is missing below the shoulder (Figure 4(5)) (Specimen 2023YXBP⑤:8: argillaceous gray pottery). A groove is present inside the lip, and it has a contracted neck and a sloping shoulder but is missing below the shoulder. The shoulder is decorated with a square pattern (Figure 4(6)) (Specimen 2023YXBP⑤:14: argillaceous gray pottery). A groove is present on the surface of the square lip, and it has a contracted neck, a round shoulder, and a plain surface; it is missing below the shoulder (Figure 4(7)).

One basin: Specimen 2023YXBP⑤:1: argillaceous gray pottery: It has a flaring mouth, a folded rim, a round lip with an inner groove, an oblique straight belly, and a plain surface but is missing below the belly (Figure 4(1)). One bowl: Specimen 2023YXBP⑤:10: argillaceous gray pottery: It has an open mouth, a square lip with a groove on the lip surface, an oblique belly, and a plain surface but is missing below the belly (Figure 4(2)). One ding leg: Argillaceous gray pottery: It has a side-triangular flat leg with a solid foot root. The tip of the foot is broken. A depression is visible on the upper part of the outer side of the leg (Figure 4(8)).

3. Likou Site

The Likou Site is located in Likou Village, Houling Community. The Huai River lies to the south, a village to the west, and the northern and eastern areas border the Huaibei City of Anhui Province. It is situated on a high terrace surrounded by water on two sides. The site measures about 300 m in length and width, covering an area of approximately 90,000 square meters, with cultural deposits ranging from 0.3 to 3.8 m thick. Collected artifacts include pottery sherds, stone tools, shells, deer antlers, and animal bones. Pottery sherds mainly date back to the Yangshao, Longshan, and Shang cultures. Pottery from the Longshan Culture period is primarily argillaceous gray pottery, along with polished black pottery, argillaceous brown pottery, and small quantities of sand-tempered gray/brown pottery and shell-tempered gray/brown pottery. The decorations include basket, square, and cord-marked patterns, with minor plain surfaces and scattered string patterns. Vessel types include storage jars, urns, basins, bowls, pottery lids (a cover for containers), and Ding.

A total of five specimens were selected for testing, including urns, storage jars, and a pottery lid.

**Figure 5:**
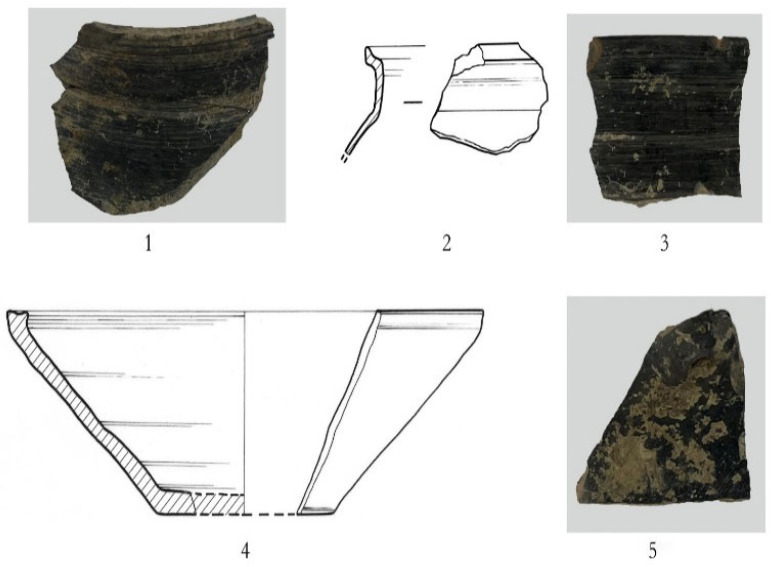
Pottery samples from the Longshan Culture period at the Likou Site. (**1**) Urn (2023YHL⑥:1), (**2**) urn (2023YHL⑥:2), (**3**) rim of a storage jar (B6:4), (**4**) pottery lid (2023YHLH2:2), and (**5**) body sherd of a storage jar (B6:3).

Two urns, both made of argillaceous black pottery, have straight mouths and sharp lips. Specimen 2023YHL⑥:1: The outer side of the rim has two raised ridges. It has a short neck and a sloping shoulder but is missing below the shoulder. The surface is plain and polished (Figure 5(1)). Specimen 2023YHL⑥:2: The inner side of the rim has a groove; it has a tall neck and a sloping shoulder but is missing below the shoulder (Figure 5(2)).

One pottery lid: Specimen 2023YHLH2:2: argillaceous gray pottery: It has an open mouth with a slightly constricted rim, a square lip with a groove on the lip surface, an oblique straight belly, and a flat base, along with wheel marks on the inside and a plain surface (Figure 5(4)).

Two storage jars are made of argillaceous black pottery with plain polished surfaces: Specimen B6:3: a body sherd (Figure 5(5)). Specimen B6:4: a flaring mouth, a rounded sharp lip, a contracted neck, and missing below the neck (Figure 5(3)).

## 2. Experimental

### 2.1. Sample Preparation

Firstly, the ceramic sample is cut into small pieces of approximately 2 cm^2^ using a cutting machine, and the sections are ground flat. Secondly, the test samples are cleaned by ultrasonic treatment in purified water twice, followed by cleaning with alcohol. For SEM-EDS analysis, the samples are broken into smaller pieces using pliers. For XRD, XRF, and IR tests, the smaller pieces were sequentially pulverized, ground, and sieved through a 0.075 mm sieve. Table 1 shows the grouping of performance index tests, including the size and number of each test and specimen.

### 2.2. Energy-Dispersive X-Ray Fluorescence Analysis

Energy-dispersive X-ray fluorescence (XRF) (Shimadzu EDX-8100, Kyoto, Japan) was used to test the chemical compositions of the bodies and surface coatings, with an X-ray spot diameter of 1.2 mm, an X-ray tube voltage of 30 kV, an X-ray tube current of 0.029 mA, and a data acquisition time of 100 s. The standard sample is Corning Glass D, which is used to calibrate the content of the samples.

### 2.3. Infrared Spectroscopy Analysis

An infrared spectrometer (XploRA PLUS, Horiba, Longjumeau, France) was used to analyze the phase of surface coatings. Infrared spectra were collected at room temperature under the 785 nm excitation line in the 100–4000 cm^−1^ spectral range. The laser beam was focused by a 50× objective lens with a laser spot diameter of 1 μm and a laser power of 1 mW.

### 2.4. X-Ray Diffraction Analysis

To use the Japanese physical X-ray diffractometer (Shimadzu Corporation, Kyoto, Japan), we took the sample after drying and grinding treatment and placed it into the glass groove for testing. The sampling interval was 0.04° (2θ); the sampling speed was 2 °/min, and the scanning angle range was 5–70° (2θ).

### 2.5. Scanning Electron Microscopy Test

The microstructure of the samples was observed using a Sigma 300 field emission environmental scanning electron microscope (Carl Zeiss AG, Oberkochen, Germany). The signal encompassed secondary electrons and backscattered electrons; the acceleration voltage was 15 kV; the vacuum level was maintained at 1 Pa; the amplification range was 18–30,000 times, and the maximum resolution was 3 nm. A thin layer of gold was deposited on the samples to enhance their conductivity for scanning electron microscopy. Backscattered electron images were obtained at a voltage of 15 kV. EDS was performed at 20 kV. The samples analyzed by SEM-EDS were prepared as polished resin blocks. The results were obtained from a single bulk chemical analysis.

## 3. Results and Discussion

### 3.1. Analysis of Chemical Composition Result

A total of 28 pottery samples were analyzed in this study, including 15 pieces from the Dazhuzhuang Site in Yongcheng, 5 pieces from the Likou Site in Yongcheng, and 8 pieces from the Biting Site in Yongcheng, all dating to the Longshan Culture period of the Neolithic Age. The 15 samples from the Dazhuzhuang Site include vessel types such as storage jars, Yan legs (tripod leg of “Yan,” a ritual cooking vessel with hollow legs), handles (“pan”, horizontal non-perforated clay attachments for gripping), Ding legs, cups, and basins, with the majority made of argillaceous gray pottery and a small number made of argillaceous brown pottery. The five samples from the Likou Site consist of pottery lids, urns, and body sherds and are mainly composed of argillaceous black pottery. The eight samples from the Biting Site include basins, bowls, storage jars, and Ding vessels, all made of argillaceous gray pottery.

From a typological perspective, these samples exhibit distinct regional characteristics. Artifacts from the Dazhuzhuang Site display a rich diversity of vessel types, covering these major functional categories, including cooking (Ding legs and Yan legs), storage (storage jars and basins), and drinking (cups), possibly reflecting the multifunctionality of the settlement. Artifacts from the Likou Site are predominantly storage vessels, such as pottery lids, urns, and body sherds, made from thin-walled, polished black pottery. In contrast, artifacts from the Biting Site are mainly utilitarian vessels for daily use, such as basins, bowls, storage jars, and Ding vessels, characterized by regular forms and consistent body structures.

#### 3.1.1. Dazhuzhuang Site

XRF was used to measure the chemical composition of 16 elements in the pottery samples unearthed from the Dazhuzhuang site. The contents of 10 elements, including SiO_2_, Al_2_O_3_, Fe_2_O_3_, K_2_O, MgO, CaO, Na_2_O, TiO_2_, and P_2_O_5_, are shown in Table 2. The detailed statistical results of chemical composition are shown in Table 3.

According to Table 3, the SiO_2_ content at the Dazhuzhuang Site ranges from 64.98% to 71.07%, with an average of 64.98% and a standard deviation of 1.19%. The Al_2_O_3_ content ranges from 15.18% to 18.83%, with an average of 16.96% and a standard deviation of 0.78%. The Fe_2_O_3_ content varies between 5.08% and 7.07%, with an average of 5.74% and a standard deviation of 0.58%. The K_2_O content ranges from 2.90% to 3.77%, with an average of 3.49% and a standard deviation of 0.26%. The MgO content ranges from 1.33% to 1.80%, with an average of 1.60% and a standard deviation of 0.11%. The CaO content varies between 1.19% and 1.81%, with an average of 1.49% and a standard deviation of 0.16%. The Na_2_O content ranges from 0.95% to 1.31%, with an average of 1.14% and a standard deviation of 0.08%.

In terms of oxide composition, the SiO_2_/Al_2_O_3_ ratio of 3.96 at the Dazhuzhuang Site indicates the use of clay with moderate weathering. The relatively high Fe_2_O_3_ content (5.74%) suggests that the raw materials may contain a significant amount of hematite, while the stable alkali metal content indicates that illite is the dominant clay mineral. Notably, SiO_2_ exhibits the largest standard deviation (1.19%) among the oxides, reflecting the most significant variability in silicon content. This variability may be attributed to the following technical characteristics in raw material processing: (1) an uneven distribution of quartz particles within the raw materials, (2) relatively lenient selection criteria for siliceous materials by local potters, and (3) the potential mixing of raw materials from different batches or sources. In contrast, the relatively stable Al_2_O_3_ content (standard deviation of 0.78%) suggests that potters in this region likely prioritized stricter quality control for clay components, while showing greater flexibility in the selection of siliceous temper materials.

#### 3.1.2. Likou Site

The chemical compositions of 16 elements in pottery samples excavated from the Likou Site were measured using X-ray fluorescence (XRF). Among these, the contents of ten elements, including SiO_2_, Al_2_O_3_, Fe_2_O_3_, K_2_O, MgO, CaO, Na_2_O, TiO_2_, and P_2_O_3_, are presented in Table 4, with detailed statistical results of their chemical compositions provided in Table 5.

As shown in Table 5, the SiO_2_ content at the Likou Site in the Yongcheng region ranges from 65.93% to 67.97%, with an average of 67.10% and a standard deviation of 0.68%. The Al_2_O_3_ content varies between 17.37% and 18.11%, averaging 17.78% (standard deviation: 0.23%). Fe_2_O_3_ levels span 5.02–6.26%, with a mean value of 5.69% and a standard deviation of 0.45%. The K_2_O content ranges from 3.01% to 3.60% (average: 3.39%; SD: 0.20%), while MgO concentrations fall within 1.58–1.91% (average: 1.71%; SD: 0.10%). CaO values are recorded between 1.46% and 2.22% (mean: 1.75%; SD: 0.28%), and the Na_2_O content ranges from 1.06% to 1.27% (average: 1.15%; SD: 0.06%). Regarding major elemental characteristics, the high Al_2_O_3_ content (17.78%) at the Likou Site suggests using high-quality kaolin or well-weathered sedimentary clay in pottery production. The SiO_2_/Al_2_O_3_ ratio of 3.77 aligns with typical clay ranges, effectively excluding the possibility of primary loess utilization.

Compared to the Dazhuzhuang site, the pottery from the Likou site exhibits significant technological differences. Firstly, in terms of elemental composition, although the levels of SiO_2_ and Fe_2_O_3_ are similar, the contents of Al_2_O_3_ and CaO are notably higher. Secondly, regarding process control, the standard deviations of Fe_2_O_3_, SiO_2_, and Al_2_O_3_ are significantly lower than those at the Dazhuzhuang site, whereas the standard deviation of CaO is relatively higher. This phenomenon may reflect the following technological characteristics during the firing process of the pottery: (1) The Likou site used high-quality clay materials with higher Al_2_O_3_ content. (2) Potters in the region demonstrated more standardized selection and processing of silico-aluminous raw materials. (3) The greater fluctuation in CaO content may indicate the intentional addition of calcareous tempering agents (such as shell powder or lime) during firing or could result from natural variations in raw material batches.

#### 3.1.3. Biting Site

The chemical composition of 16 elements in the pottery samples unearthed from the Biting site was measured using X-ray fluorescence (XRF). Among them, the contents of 10 major elements—SiO_2_, Al_2_O_3_, Fe_2_O_3_, K_2_O, MgO, CaO, Na_2_O, TiO_2_, and P_2_O_5_—are presented in Table 6. Detailed statistical results of the chemical composition are shown in Table 7.

As shown in Table 7, the SiO_2_ content in pottery from the Biting site in the Yongcheng area ranges from 65.19% to 69.01%, with an average of 67.29% and a standard deviation of 1.30%. The Al_2_O_3_ content ranges from 15.88% to 18.08%, with an average of 17.36% and a standard deviation of 0.62%. Fe_2_O_3_ ranges from 5.28% to 7.11%, averaging 5.98% with a standard deviation of 0.59%. The K_2_O content falls between 3.11% and 4.32%, with a mean of 3.49% and a standard deviation of 0.34%. MgO ranges from 1.41% to 1.98%, averaging 1.72% with a standard deviation of 0.19%. CaO ranges from 0.84% to 1.81%, with an average of 1.41% and a standard deviation of 0.28%. The Na_2_O content ranges from 0.96% to 1.52%, with a mean of 1.25% and a standard deviation of 0.16%. Regarding major elements, the high Al_2_O_3_ content (up to 17.78%) in pottery from the Biting site suggests the use of high-quality kaolinite or well-weathered sedimentary clay as raw materials. The SiO_2_/Al_2_O_3_ ratio of 3.87 indicates a high degree of weathering of the raw materials. The notable Na_2_O content (1.25%) may reflect the presence of sodium feldspar minerals in the clay.

Compared to the Dazhuzhuang and Likou Sites, the pottery from the Biting Site exhibits the highest Al_2_O_3_ content and the lowest CaO content, although with greater variability. In contrast, the distribution of SiO_2_ content is the most stable, suggesting that calcareous tempering materials were rarely used during the firing process.

### 3.2. Two-Dimensional Scatter Analysis of XRF Data

In archeological science research, two-dimensional scatter analysis of elemental concentrations is important for revealing the characteristics of raw materials used in ancient artifacts. Based on the chemical composition of the pottery, this study selects four major oxides—SiO_2_, Al_2_O_3_, CaO, and Fe_2_O_3_—for two-dimensional scatter analysis [47].

**Figure 6:**
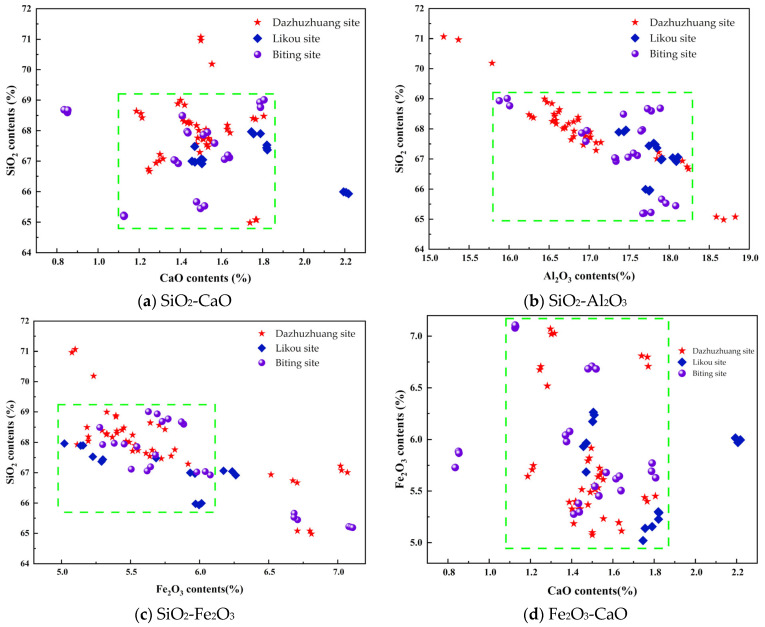
Two-dimensional compositional analysis of oxides in pottery unearthed from the Dazhuzhuang, Likou, and Biting Sites in the Yongcheng District.

Figure 6a presents a two-dimensional scatter plot of the SiO_2_ and CaO content. As shown in the figure, the pottery unearthed from the Dazhuzhuang, Likou, and Biting Sites in the Yongcheng area generally has SiO_2_ concentrations between 65.00% and 69.00% and CaO concentrations mostly ranging from 1.10% to 1.85%. The SiO_2_/CaO ratios vary widely, ranging from 35.14 to 62.72. Specifically, while the CaO content at the Dazhuzhuang site is relatively concentrated, the SiO_2_ distribution is significantly more scattered than at the other two sites, with a standard deviation of up to 1.19. In contrast, artifacts from the Biting Site generally show lower CaO content, whereas those from the Likou Site display higher CaO levels.

Figure 6b illustrates the bivariate distribution of SiO_2_ and Al_2_O_3_ for pottery from the three sites. The analysis reveals that most samples from Dazhuzhuang, Likou, and Biting fall within the ranges of 65.00–69.00% for SiO_2_ and 15.70–18.25% for Al_2_O_3_. The SiO_2_/Al_2_O_3_ ratios range from 3.56 to 4.40, which is significantly lower than the typical 7–10 range found in the loess of the middle Yellow River region [48]. According to geochemical indicators proposed by Liu Dongsheng [49], such low ratios may reflect the following formation mechanisms: (1) The pottery raw materials were derived from highly weathered sediments. (2) The materials formed under humid climatic conditions and underwent intense chemical weathering. (3) There may have been deliberate human processes of raw material selection or levigation. Notably, the three sites exhibit different distribution patterns. Dazhuzhuang shows the greatest elemental dispersion, possibly due to varied raw material sources or processing methods. In contrast, Likou shows the most concentrated distribution, which, along with its significantly larger site scale, indirectly suggests a more standardized raw material selection and processing system. These differences provide important clues for understanding ceramic technological traditions in the Neolithic settlements of the Yongcheng region and offer new perspectives for exploring the relationship between settlement size, hierarchy, and craft production in the Neolithic period.

Figure 6c presents a two-dimensional scatter plot of the SiO_2_ and Fe_2_O_3_ content. The pottery from all three sites shows SiO_2_ concentrations mainly between 65.00% and 69.00%, and Fe_2_O_3_ concentrations between 5.00% and 6.00%, with SiO_2_/Fe_2_O_3_ ratios mostly in the range of 11.5–13. Among them, Dazhuzhuang shows the greatest dispersion in both SiO_2_ and Fe_2_O_3_ contents, while Likou and Biting show more concentrated SiO_2_ distributions, though the Fe_2_O_3_ content still varies considerably.

Figure 6d shows the two-dimensional scatter plot of the Fe_2_O_3_ and CaO content. Pottery from all three sites generally has Fe_2_O_3_ concentrations between 5.00% and 6.00% and CaO concentrations between 1.10% and 1.85%, with Fe_2_O_3_/CaO ratios ranging from 2.70 to 5.45. From the figure, it can be seen that the Fe_2_O_3_ content at Dazhuzhuang is more widely dispersed compared to Likou and Biting. In contrast, the CaO content is more tightly clustered, indicating that potters at Dazhuzhuang had stricter control over calcareous components in their raw materials.

In summary, although the Dazhuzhuang, Likou, and Biting Sites are all located in the Yongcheng area of Shangqiu and their pottery dates to the Longshan period, two-dimensional scatter analysis of elemental composition reveals notable differences. These distribution patterns reflect the diversity of ceramic raw materials in the region and suggest differentiated strategies of raw material selection or technological traditions across the sites. Dazhuzhuang, in particular, exhibits the highest degree of elemental variability, indicating a relatively loose standard for raw material selection—likely involving the mixing of various clays or sourcing materials from multiple locations. Notably, no kiln remains have been discovered at Dazhuzhuang despite a surveyed site area of 16,800 square meters. Whether a site of this scale during the Longshan culture possessed the technology and facilities for independent pottery production remains a topic for further investigation.

### 3.3. Microcomposition-IR

**Figure 7:**
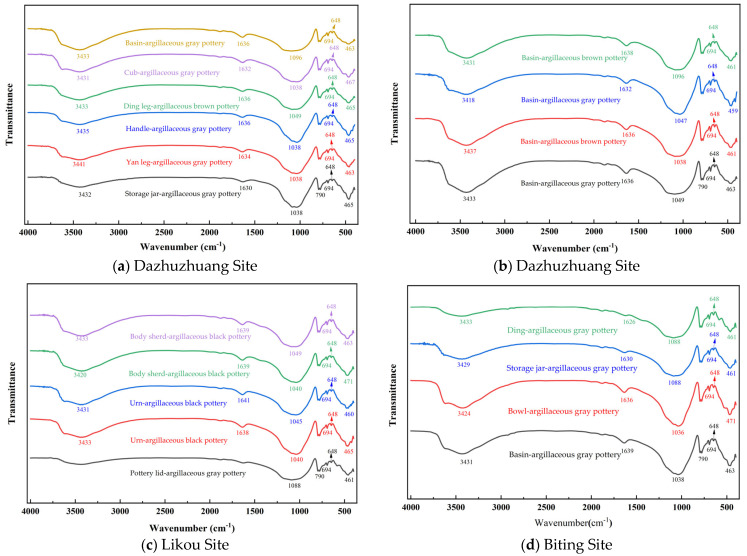
Infrared spectral analysis of pottery artifacts unearthed from the Dazhuzhuang, Likou, and Biting Sites in the Yongcheng area.

Infrared (IR) analysis was conducted on pottery artifacts unearthed from the Dazhuzhuang, Likou, and Biting Sites in the Yongcheng area, with the results shown in Figure 4. Figure 7a,b display the IR spectra of samples from the Dazhuzhuang Site; Figure 7c presents the spectrum from the Likou Site, and Figure 7d shows the spectrum from the Biting Site. The analysis of Figure 7a,b reveals that the IR spectra of artifacts from the Dazhuzhuang Site exhibit notable heterogeneity. Under identical conditions, there are fluctuations in the intensity of major absorption peaks and the widths of absorption bands, indicating a diversity in raw material sources. As shown in Figure 7c, the argillaceous black pottery from the Likou Site exhibits a pronounced absorption trough near 3400 cm^−1^, while the argillaceous gray pottery also presents a distinct absorption band in the same region. This feature is typically attributed to the stretching vibrations of O–H bonds, which likely originate from the presence of adsorbed or structural water within the materials. Further analysis of the IR data from the Biting Site (Figure 7d) shows that some distinctions are evident, while peak positions across different vessel types largely coincide. Jars and tripods exhibit stronger peaks at around 1000 cm^−1^, whereas basins and bowls show weaker peaks at the same position, but with significantly broadened absorption bands. These variations reflect differences in raw material composition, forming techniques, and thermal conditions tailored to the functional requirements of different vessel types [50].

### 3.4. Phase Analysis Using XRD

**Figure 8:**
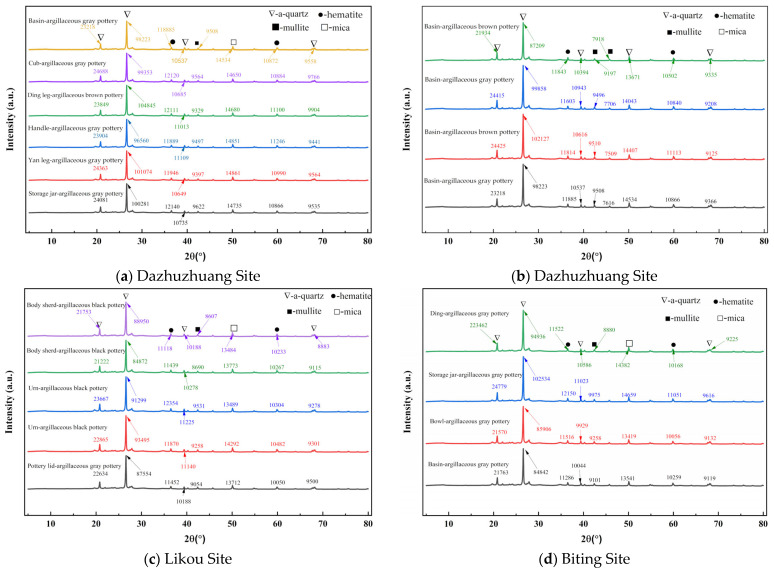
XRD pattern analysis of pottery artifacts unearthed from the Dazhuzhuang, Likou, and Biting Sites in the Yongcheng area.

XRD analysis was conducted on pottery artifacts unearthed from the Dazhuzhuang, Likou, and Biting Sites in the Yongcheng area, with the results shown in Figure 8. Figure 8a,b display the XRD spectra of samples from the Dazhuzhuang Site; Figure 8c presents data from the Likou Site, and Figure 8d shows results from the Biting Site. The interpretation of the XRD spectra indicates a high degree of consistency in the primary mineral composition of pottery from all three sites. Common characteristic phases include mica (KAl_2_(AlSi_3_O_10_)(OH)_2_), mullite (3Al_2_O_3_·2SiO_2_), quartz (SiO_2_), and hematite (Fe_2_O_3_). Kaolinite, which was a key raw material for ceramic bodies in northern China, undergoes significant phase transformations during firing: At 450–650 °C, it loses structural water (Al_2_Si_2_O_5_(OH)_4_ → Al_2_Si_2_O_7_ + 2H_2_O), forming amorphous metakaolinite. Beyond 900 °C, metakaolinite decomposes into free Al_2_O_3_ and SiO_2_ (Al_2_Si_2_O_7_ → Al_2_O_3_ + 2SiO_2_) and recrystallizes into mullite around 1100 °C, with excess SiO_2_ remaining as quartz. The pronounced intensity of quartz peaks in the XRD spectra may stem from crystallized free SiO_2_ from the thermal decomposition of kaolinite and the partial transformation of original quartz particles. Although the characteristic peaks of mullite are relatively weak, the combined presence of mullite and quartz forms the ceramic skeleton structure [51]. The residual presence of hematite further confirms that firing temperatures commonly exceeded 900 °C [52]. This aligns with the advanced high-temperature firing techniques of the Late Neolithic period in the Yellow River basin.

Figure 8a,b show that due to the heterogeneous nature of raw materials used at the Dazhuzhuang Site, no clear patterns emerge in the XRD spectra. In Figure 8c, the clayey black pottery sample from the Likou Site exhibits a significantly stronger quartz peak at 2θ = 26.6° compared to contemporaneous gray pottery. Generally, black pottery shows stronger quartz peaks than gray pottery. Notably, different vessel types also show variability; urns have stronger quartz peaks than body sherds. Further analysis of the XRD data from the Biting Site, as shown in Figure 8d, reveals that under similar clayey gray pottery conditions, jars exhibit distinct phase characteristics: (1) Quartz peaks (2θ = 26.6°) are significantly stronger than those of tripods, bowls, and basins. (2) The intensity of the hematite peak (2θ = 33.2°) also increases. (3) A clear positive correlation is observed between the intensities of quartz and hematite peaks. These findings suggest that as representative storage vessels, jars may have incorporated more quartz temper to enhance their mechanical strength. Meanwhile, the elevated hematite content could be associated with specific surface treatment techniques used for jars, which may improve their density and extend their service life.

In conclusion, XRD phase analysis of pottery from the Dazhuzhuang, Likou, and Biting Sites reveals important characteristics of ceramic production techniques. All three sites had mastered kiln technologies capable of reaching high temperatures (900–1100 °C). The residual hematite peak at 2θ = 33.2° suggests that oxidizing atmospheres were deliberately maintained during firing, implying that temperature control techniques were likely adapted to suit different vessel types. The preservation of the mica phase (2θ = 8.8°) indicates that the firing temperature did not reach full vitrification levels (<1200 °C).

### 3.5. Microstructure Analysis via SEM

#### 3.5.1. Dazhuzhuang Site

**Figure 9:**
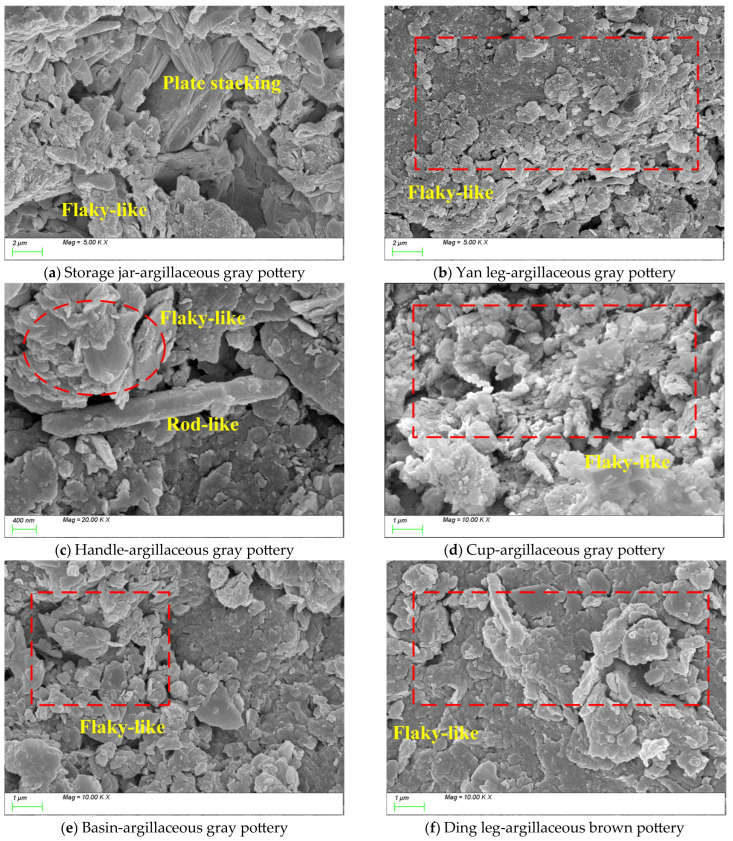
SEM images of pottery sherds unearthed from the Dazhuzhuang Site.

Figure 9 presents SEM images of pottery from the Dazhuzhuang Site. The object types from Figure 9a–e are a storage jar, a Yan leg, a handle, a cup, a basin, and a ding leg, respectively. In terms of pottery fabrics, except for the ding leg, which is made of argillaceous brown pottery, all other samples are made of argillaceous gray pottery. From Figure 9a–f, it can be observed that at the level of the clay matrix, clay aggregates and partially vitrified clay platelets are commonly present within the matrix, displaying microstructural characteristics typical of the Longshan Culture. Notable technological variations are evident among different vessel types. In the argillaceous gray pottery samples (Figure 9a–e), the clay platelets generally exhibit layered accumulation yet show vessel-specific differences: The storage jar (Figure 9a) exhibits a typical heterogeneous structure with numerous partially vitrified clay platelets (5–15 μm) and aggregates (20–50 μm in diameter), suggesting a relatively low firing temperature [53]. The Yan leg (Figure 9b) shows unevenly distributed and loosely packed inclusions, which may be related to functional adjustments in manufacturing. The handle (Figure 9c) exhibits not only the layered platelet structure but also a small number of rod-shaped features, likely mullite whiskers formed at high temperatures (>1000 °C), indicating targeted high-temperature treatment for this part. The cup (Figure 9d) displays well-developed clay mineral growth with denser layered structures. The basin (Figure 9e) and the ding leg (Figure 9f) possess orderly layered structures and uniformly distributed inclusions. Although the argillaceous brown pottery ding leg differs chemically from the argillaceous gray pottery basin, their microstructures are highly similar. These findings suggest the following: (1) Potters at the Dazhuzhuang Site had mastered techniques to adjust manufacturing processes according to the functional requirements of different vessel types. (2) The appearance of mullite whiskers indicates that localized high-temperature treatments were employed to enhance the mechanical properties of critical components. (3) Despite differences in chemical composition, argillaceous brown pottery and gray pottery likely shared similar forming techniques, with variations mainly in firing atmosphere control.

#### 3.5.2. Likou Site

**Figure 10:**
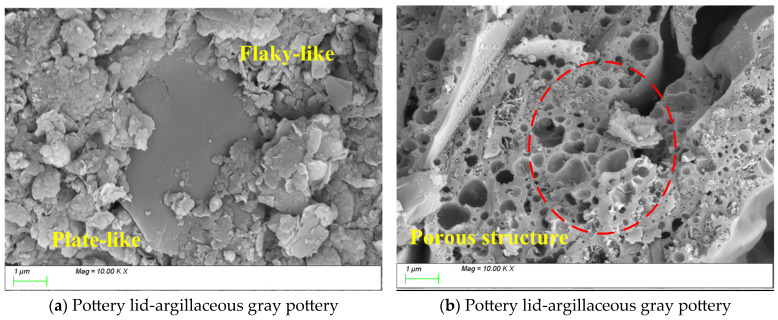
An SEM image of a pottery lid unearthed from the Likou Site.

**Figure 11:**
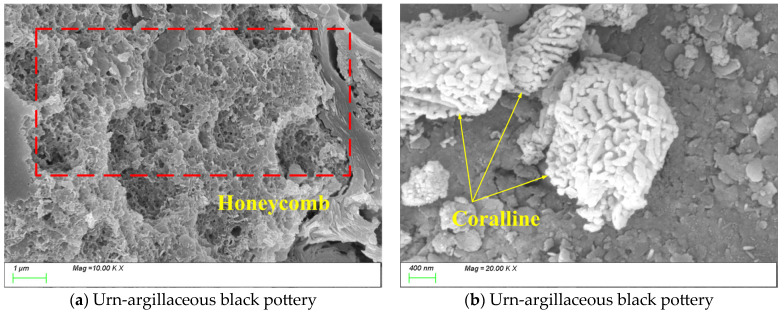
An SEM image of an urn unearthed from the Likou Site.

**Figure 12:**
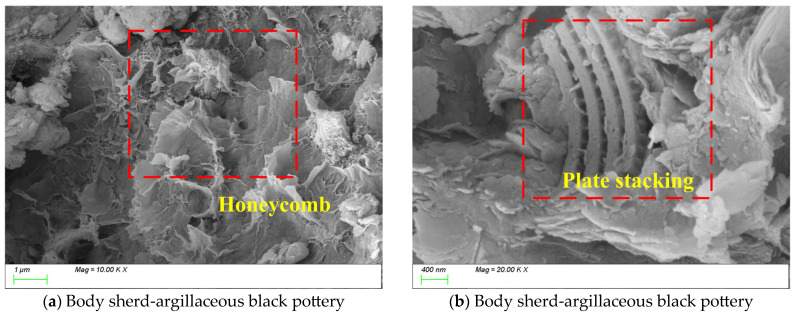
SEM image of a body sherd unearthed from the Likou Site.

Figure 10, Figure 11 and Figure 12 present SEM images of pottery sherds excavated from the Likou Site. Figure 7 shows a sample identified as a pottery lid that is made of argillaceous gray pottery. A comparative analysis reveals significant differences in the microstructural characteristics of the artifacts from the Likou Site compared to those from Dazhuzhuang Site. As shown in Figure 10a, although similar to Dazhuzhuang in exhibiting flaky and platy structures, the pottery lid from Likou displays a notable decrease in quantity but an increase in the size of the platy phases. The appearance of these large platy structures suggests that the clay minerals used as raw materials had a higher degree of crystallinity and that the firing process was controlled more precisely [54], potentially involving specialized forming techniques. In Figure 10b, the matrix predominantly exhibits a porous structure, with significant variability in pore size distribution. Combined with the elevated CaO content identified in the compositional analysis, it is inferred that a calcium-based flux was intentionally added during the firing process to promote foaming and enhance porosity [55].

The vessel type in Figure 11 is an urn that is made of argillaceous black pottery. Microstructural and characteristic phase analysis of Figure 11 reveals that while the urn shares a porous structure similar to that of the gray pottery storage jar, it exhibits more densely vitrified regions with fewer pores (Figure 11a). As shown in Figure 11b, coral-like accumulations are observed within the matrix. Energy-dispersive spectroscopy (EDS) analysis indicates that the Fe content in the coral-like regions (8–10%) is significantly higher than that of the surrounding matrix (5–6%), confirming a segregation effect of iron. The formation mechanisms of these special coral-like structures may involve (1) phase separation and recrystallization of iron-bearing minerals at high temperatures, (2) compositional segregation within the melt, and (3) non-equilibrium solidification induced by specific cooling rates.

The vessel type in Figure 12 is a body sherd from a pottery storage jar that is made of argillaceous black pottery. As shown in Figure 12a, the matrix structure exhibits a honeycomb-like growth pattern. Figure 12b reveals the presence of numerous plate-like products with a preferred orientation within the matrix. Energy-dispersive spectroscopy (EDS) analysis indicates that the plate-like phases are rich in Al_2_O_3_ (22 ± 2%) and exhibit a dense structure [56], while the surrounding matrix is primarily composed of SiO_2_ (68 ± 3%). This compositional differentiation at the microscale may result from (1) preferentially oriented growth of kaolinite-derived phases at high temperatures, (2) phase separation induced by compositional gradients in the melt, and (3) non-equilibrium crystallization triggered by controlled cooling processes.

In summary, comparative analysis indicates that compared to argillaceous gray pottery, argillaceous black pottery exhibits more densely vitrified regions, fewer pores, and a reduced pore size distribution. These structural features confer improved mechanical strength and thermal stability to the black pottery. Collectively, the observed microstructural characteristics suggest that the production of black pottery at the Likou Site involved sophisticated firing control techniques, reflecting a highly advanced material design achieved during the late Longshan Culture period.

#### 3.5.3. Biting Site

**Figure 13:**
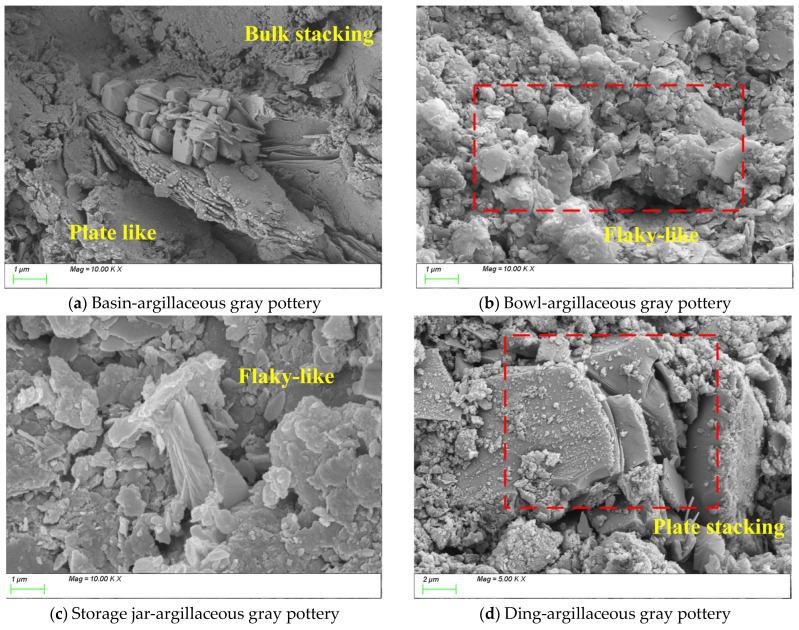
SEM image of pottery unearthed from the Biting Site.

Figure 13 presents SEM images of pottery from the Biting Site. The vessel types from Figure 13a–d are a basin, a bowl, a storage jar, and a Ding, all made of argillaceous gray pottery. Although all samples are classified as argillaceous gray pottery, significant technological differences are observed among different vessel types at the microstructural level [57]. The basin (Figure 13a) exhibits a distinctive “block-plate” synergistic accumulation structure, where blocky phases and thin plate-like phases interlock to form a network. The bowl (Figure 13b) displays a typical clay platelet stacking pattern with moderate porosity, balancing requirements for lightweight construction and thermal insulation. The storage jar (Figure 13c) also shows a platelet stacking structure but with a denser arrangement and a high degree of platelet orientation, which is favorable for enhancing mechanical strength and thermal stability. In contrast, the Ding (Figure 13d) presents the densest matrix structure, where well-developed plate-like products form strong interfacial bonding with the matrix. The extensive growth and tight integration of the plate-like phases contribute significantly to improved mechanical properties and thermal performance.

In summary, these microstructural differences indirectly reflect the potters’ profound understanding of the functional requirements of different vessel types and their mastery of targeted raw material preparation and forming techniques. Notably, the densification observed in the storage jar and ding indicates specialized technological adaptations for cooking and storage vessels. In contrast, the relatively more porous structures of the bowl and basin reflect different functional priorities for daily use items. This precise matching of material properties, functional demands, and technological choices demonstrates an advanced understanding of performance optimization and reveals a highly developed capability to systematically integrate material characteristics with vessel functions. From an archeological perspective, such a highly specialized technical system marks the transition of Neolithic pottery production from empirical practices to a more scientific approach, providing a significant example for understanding the technological evolution of prehistoric craft industries.

## 4. Conclusions

This study conducted a comprehensive multidisciplinary analysis of pottery unearthed from three Longshan Culture sites—Dazhuzhuang, Likou, and Biting—in the Yongcheng District of Henan Province. By integrating modern analytical techniques such as X-ray fluorescence (XRF), X-ray diffraction (XRD), infrared spectroscopy (IR), and scanning electron microscopy with energy-dispersive spectroscopy (SEM-EDS), together with archeological typology and materials science approaches, the following major conclusions were drawn:

(1) Raw Material Sources:

Chemical composition analysis reveals variations in raw material selection among the sites. The pottery from the Dazhuzhuang Site exhibits significant fluctuations in SiO_2_ content, indicating flexible raw material choices. The Likou Site pottery shows high Al_2_O_3_ and CaO content, suggesting the use of high-quality kaolinite clay and the addition of calcium-based fluxes. In contrast, the Biting Site pottery displays more weathered raw materials and the lowest CaO content among the three sites.

(2) Technological Aspects:

Pottery from the Dazhuzhuang Site, predominantly argillaceous gray pottery, exhibits an uneven distribution of clay platelets and aggregates at the microstructural level, indicating relatively low firing temperatures (around 900 °C) and less controlled manufacturing processes. The Likou Site pottery, primarily argillaceous black pottery, features dense vitrified structures and iron segregation phenomena, suggesting the application of high-temperature reducing atmospheres and a high degree of technological standardization. At the Biting Site, the vessel-specific microstructural variations (e.g., dense laminar arrangements in storage jars) reflect precise adjustments in raw material preparation and firing techniques tailored to the functional demands of different pottery forms.

(3) Cultural Significance:

The high Al_2_O_3_ content in the black pottery from the Likou Site is comparable to that of the Shandong Longshan Culture, implying possible technological interactions between regions. In contrast, the gray pottery from the Dazhuzhuang and Biting Sites reflects localized production traditions.

(4) Research Implications:

This study highlights the technological diversity and complexity of pottery production in the Yongcheng area during the Longshan period. It provides critical empirical evidence for exploring the technological evolution, raw material strategies, and functional adaptations of late Neolithic craft industries. Future research could benefit from comparative studies across broader site distributions and incorporating advanced techniques, such as stable isotope tracing, to identify clay sources accurately. Ultimately, reconstructing ancient pottery production chains will enable a more comprehensive understanding of the technological evolution of prehistoric crafts and their role in early social complexity and civilization development. Advancing these research directions will help uncover the intrinsic mechanisms of technological innovation and its significance in the origins of early civilizations.

## Figures and Tables

**Table 1:** Grouping of the performance index tests.

Performance Index	Specimen Size	Quantity
Energy-dispersive X-ray fluorescence (XRF)	20 mm × 20 mm × 20 mm	84
Infrared spectroscopy (IR)	20 mm × 20 mm × 20 mm	57
X-ray diffraction analysis (XRD)	20 mm × 20 mm × 20 mm	57
Scanning electron microscopy (SEM)	40 mm × 40 mm × 40 mm	49

**Table 2:** XRF experimental data results of the Dazhuzhuang Site in the Yongcheng area (content: wt.%).

Sample Number	Pottery Type	Part	SiO_2_	Al_2_O_3_	Fe_2_O_3_	K_2_O	MgO	CaO	Na_2_O	TiO_2_	P_2_O_5_
2023YPDH1:1-1	argillaceous gray pottery	Storage jar	68.41	16.28	5.44	3.52	1.63	1.75	1.30	0.74	0.63
2023YPDH1:1-2	argillaceous gray pottery	Storage jar	68.48	16.25	5.45	3.50	1.57	1.81	1.24	0.75	0.66
2023YPDH1:1-3	argillaceous gray pottery	Storage jar	68.37	16.31	5.40	3.54	1.59	1.77	1.31	0.75	0.68
2023YPDH1:23-1	argillaceous gray pottery	Yan leg	68.01	16.67	5.49	3.70	1.67	1.49	1.15	0.76	0.77
2023YPDH1:23-2	argillaceous gray pottery	Yan leg	68.04	16.69	5.47	3.66	1.68	1.53	1.13	0.75	0.79
2023YPDH1:23-3	argillaceous gray pottery	Yan leg	68.17	16.59	5.37	3.72	1.69	1.48	1.20	0.75	0.77
2023YPDH1:17-1	argillaceous gray pottery	Handle	68.25	16.54	5.33	3.66	1.63	1.44	1.16	0.74	0.98
2023YPDH1:17-2	argillaceous gray pottery	Handle	68.50	16.56	5.19	3.58	1.71	1.41	1.11	0.73	0.98
2023YPDH1:17-3	argillaceous gray pottery	Handle	68.29	16.58	5.33	3.66	1.64	1.44	1.10	0.74	0.98
2023YPDH1:21-1	argillaceous brown pottery	Ding leg	68.24	16.81	5.52	3.57	1.60	1.45	1.16	0.74	0.65
2023YPDH1:21-2	argillaceous brown pottery	Ding leg	68.30	16.87	5.40	3.55	1.61	1.42	1.16	0.73	0.72
2023YPDH1:21-3	argillaceous brown pottery	Ding leg	68.39	16.86	5.29	3.51	1.63	1.41	1.12	0.74	0.83
2023YPDH1:9-1	argillaceous brown pottery	Storage jar	68.84	16.54	5.40	3.60	1.63	1.42	1.13	0.73	0.55
2023YPDH1:9-2	argillaceous brown pottery	Storage jar	68.88	16.47	5.39	3.62	1.65	1.39	1.14	0.73	0.55
2023YPDH1:9-3	argillaceous brown pottery	Storage jar	69.00	16.44	5.33	3.62	1.63	1.40	1.14	0.74	0.53
2023YPDH1:4-1	argillaceous gray pottery	Storage jar	67.29	17.09	5.92	3.62	1.65	1.50	1.29	0.78	0.60
2023YPDH1:4-2	argillaceous gray pottery	Storage jar	67.76	16.95	5.82	3.60	1.62	1.49	1.22	0.77	0.54
2023YPDH1:4-3	argillaceous gray pottery	Storage jar	67.55	17.15	5.79	3.64	1.63	1.48	1.23	0.76	0.54
2023YPDH1:5-1	argillaceous gray pottery	Storage jar	67.93	16.81	5.11	3.68	1.62	1.64	1.12	0.74	1.10
2023YPDH1:5-2	argillaceous gray pottery	Storage jar	68.18	16.74	5.20	3.50	1.61	1.63	1.09	0.74	1.06
2023YPDH1:5-3	argillaceous gray pottery	Storage jar	68.05	16.70	5.19	3.65	1.59	1.63	1.13	0.74	1.08
2023YPDH1:19-1	argillaceous gray pottery	Cub	68.64	16.63	5.64	3.42	1.80	1.19	1.10	0.71	0.65
2023YPDH1:19-2	argillaceous gray pottery	Cub	68.56	16.62	5.71	3.51	1.74	1.21	1.11	0.74	0.61
2023YPDH1:19-3	argillaceous gray pottery	Cub	68.43	16.57	5.75	3.51	1.77	1.21	1.16	0.73	0.62
2023YPDH1:13-1	argillaceous gray pottery	Basin	67.64	16.99	5.61	3.74	1.53	1.55	1.12	0.75	0.76
2023YPDH1:13-2	argillaceous gray pottery	Basin	67.54	17.09	5.64	3.74	1.53	1.53	1.17	0.74	0.76
2023YPDH1:13-3	argillaceous gray pottery	Basin	67.90	17.01	5.53	3.67	1.49	1.53	1.08	0.73	0.84
2023YPDH1:12-1	argillaceous brown pottery	Basin	67.71	17.01	5.52	3.75	1.53	1.51	1.17	0.74	0.78
2023YPDH1:12-2	argillaceous brown pottery	Basin	67.90	16.94	5.54	3.76	1.50	1.51	1.10	0.75	0.75
2023YPDH1:12-3	argillaceous brown pottery	Basin	67.73	17.02	5.55	3.77	1.54	1.51	1.14	0.75	0.75
2023YPDH1:11-1	argillaceous gray pottery	Basin	67.47	16.93	5.72	3.71	1.59	1.54	1.07	0.73	0.96
2023YPDH1:11-2	argillaceous gray pottery	Basin	67.75	16.81	5.66	3.73	1.60	1.54	1.05	0.72	0.94
2023YPDH1:11-3	argillaceous gray pottery	Basin	67.64	16.78	5.68	3.72	1.60	1.55	1.09	0.73	0.97
2023YPDH1:16-1	argillaceous gray pottery	Storage jar	67.08	17.90	7.03	2.94	1.43	1.32	1.11	0.76	0.22
2023YPDH1:16-2	argillaceous gray pottery	Storage jar	67.01	17.85	7.07	3.00	1.43	1.30	1.10	0.77	0.27
2023YPDH1:16-3	argillaceous gray pottery	Storage jar	67.22	17.87	7.02	2.96	1.41	1.30	1.08	0.76	0.17
2023YPDH2:12-1	argillaceous brown pottery	Basin	65.08	18.59	6.80	3.35	1.68	1.77	1.00	0.79	0.69
2023YPDH2:12-2	argillaceous brown pottery	Basin	65.08	18.83	6.71	3.27	1.68	1.77	0.95	0.80	0.71
2023YPDH2:12-3	argillaceous brown pottery	Basin	64.98	18.68	6.81	3.36	1.72	1.74	1.00	0.79	0.68
2023YPDH2:1-1	argillaceous gray pottery	Storage jar	66.94	18.17	6.52	3.18	1.67	1.28	1.08	0.79	0.17
2023YPDH2:1-2	argillaceous gray pottery	Storage jar	66.74	18.22	6.68	3.20	1.68	1.25	1.04	0.80	0.18
2023YPDH2:1-3	argillaceous gray pottery	Storage jar	66.67	18.24	6.71	3.18	1.70	1.25	1.06	0.79	0.18
2023YPDH2:5-1	argillaceous gray pottery	Storage jar	70.19	15.79	5.23	2.95	1.40	1.55	1.24	0.78	0.65
2023YPDH2:5-2	argillaceous gray pottery	Storage jar	70.97	15.37	5.08	2.90	1.37	1.50	1.20	0.77	0.63
2023YPDH2:53	argillaceous gray pottery	Storage jar	71.07	15.18	5.10	2.91	1.33	1.50	1.24	0.77	0.66

**Table 3:** Statistical results of the chemical composition of the pottery shards from the Dazhuzhuang Site in the Yongcheng area (wt.%).

Statistical Measures	SiO_2_	Al_2_O_3_	Fe_2_O_3_	K_2_O	MgO	CaO	Na_2_O	TiO_2_
Number of Samples	45	45	45	45	45	45	45	45
Mean	67.93	16.96	5.74	3.49	1.60	1.49	1.14	0.75
Maximum	71.07	18.83	7.07	3.77	1.80	1.81	1.31	0.80
Minimum	64.98	15.18	5.08	2.90	1.33	1.19	0.95	0.71
Standard Deviation	1.19	0.78	0.58	0.26	0.11	0.16	0.08	0.02

**Table 4:** XRF experimental data results of the Likou Site in the Yongcheng area (content: wt.%).

Sample Number	Pottery Type	Part	SiO_2_	Al_2_O_3_	Fe_2_O_3_	K_2_O	MgO	CaO	Na_2_O	TiO_2_
2023YHLH2:2-1	argillaceous gray pottery	Pottery lid	67.06	18.11	6.17	3.01	1.70	1.50	1.22	0.82
2023YHLH2:2-2	argillaceous gray pottery	Pottery lid	67.04	18.04	6.24	3.01	1.70	1.51	1.22	0.82
2023YHLH2:2-3	argillaceous gray pottery	Pottery lid	66.91	18.09	6.26	3.03	1.70	1.51	1.27	0.81
2023YHL⑥:1-1	argillaceous black pottery	Urn	67.89	17.37	5.14	3.47	1.70	1.76	1.15	0.80
2023YHL⑥:12	argillaceous black pottery	Urn	67.90	17.43	5.16	3.43	1.69	1.79	1.10	0.80
2023YHL⑥:1-3	argillaceous black pottery	Urn	67.97	17.46	5.02	3.47	1.67	1.75	1.16	0.79
2023YHL⑥:2-1	argillaceous black pottery	Urn	67.48	17.83	5.69	3.34	1.85	1.47	1.08	0.78
2023YHL⑥:2-2	argillaceous black pottery	Urn	67.00	17.91	5.93	3.43	1.91	1.46	1.06	0.79
2023YHL⑥:2-3	argillaceous black pottery	Urn	66.97	17.90	5.97	3.44	1.87	1.47	1.09	0.80
2023YHLB6:3-1	argillaceous black pottery	Body sherd	65.93	17.75	6.00	3.60	1.71	2.22	1.12	0.82
2023YHLB6:3-2	argillaceous black pottery	Body sherd	65.99	17.70	6.01	3.59	1.70	2.19	1.14	0.82
2023YHLB6:3-3	argillaceous black pottery	Body sherd	65.97	17.76	5.97	3.60	1.70	2.21	1.12	0.83
2023YHLB6:4-1	argillaceous black pottery	Body sherd	67.44	17.75	5.30	3.46	1.58	1.82	1.20	0.80
2023YHLB6:4-2	argillaceous black pottery	Body sherd	67.37	17.85	5.29	3.45	1.58	1.82	1.19	0.80
2023YHLB6:4-3	argillaceous black pottery	Body sherd	67.53	17.81	5.23	3.49	1.59	1.82	1.09	0.80

**Table 5:** Statistical results of the chemical composition of the pottery shards from the Likou Site in the Yongcheng area (wt.%).

Statistical Measures	SiO_2_	Al_2_O_3_	Fe_2_O_3_	K_2_O	MgO	CaO	Na_2_O	TiO_2_
Number of Samples	15	15	15	15	15	15	15	15
Mean	67.10	17.78	5.69	3.39	1.71	1.75	1.15	0.81
Maximum	67.97	18.11	6.26	3.60	1.91	2.22	1.27	0.83
Minimum	65.93	17.37	5.02	3.01	1.58	1.46	1.06	0.78

**Table 6:** XRF experimental data results from the Yiting Site in the Yongcheng area (content: wt.%).

Sample Number	Pottery Type	Part	SiO_2_	Al_2_O_3_	Fe_2_O_3_	K_2_O	MgO	CaO	Na_2_O	TiO_2_	P_2_O_5_
2023YXBP⑤:1-1	argillaceous gray pottery	Basin	67.12	17.60	5.51	3.37	1.73	1.64	1.31	0.82	0.67
2023YXBP⑤:1-2	argillaceous gray pottery	Basin	67.06	17.49	5.62	3.39	1.74	1.62	1.36	0.82	0.67
2023YXBP⑤:1-3	argillaceous gray pottery	Basin	67.20	17.55	5.65	3.28	1.73	1.63	1.27	0.81	0.65
2023YXBP⑤:10-1	argillaceous gray pottery	Bowl	67.95	16.97	5.45	3.35	1.76	1.53	1.33	0.82	0.62
2023YXBP⑤:10-2	argillaceous gray pottery	Bowl	67.86	16.91	5.55	3.34	1.81	1.51	1.34	0.81	0.62
2023YXBP⑤:10-3	argillaceous gray pottery	Bowl	67.59	16.96	5.68	3.39	1.81	1.57	1.29	0.83	0.64
2023YXBP⑤:7-1	argillaceous gray pottery	Storage jar	67.97	17.67	5.38	3.43	1.50	1.43	1.28	0.82	0.29
2023YXBP⑤:7-2	argillaceous gray pottery	Storage jar	68.49	17.43	5.28	3.27	1.52	1.41	1.34	0.77	0.25
2023YXBP⑤:7-3	argillaceous gray pottery	Storage jar	67.93	17.65	5.30	3.39	1.55	1.44	1.41	0.79	0.31
2023YXBP⑤:21-1	argillaceous gray pottery	Storage jar	65.22	17.77	7.08	4.31	1.95	1.13	0.96	0.93	0.45
2023YXBP⑤:21-2	argillaceous gray pottery	Storage jar	65.20	17.69	7.10	4.28	1.98	1.13	1.04	0.90	0.45
2023YXBP⑤:21-3	argillaceous gray pottery	Storage jar	65.19	17.67	7.11	4.32	1.97	1.13	1.02	0.93	0.46
2023YXBP⑤:13-1	argillaceous gray pottery	Storage jar	67.02	17.33	5.98	3.66	1.89	1.38	1.21	0.79	0.51
2023YXBP⑤:13-2	argillaceous gray pottery	Storage jar	66.92	17.34	6.08	3.67	1.88	1.39	1.24	0.79	0.49
2023YXBP⑤:13-3	argillaceous gray pottery	Storage jar	67.04	17.32	6.04	3.66	1.88	1.37	1.16	0.79	0.51

**Table 7:** Statistical results of the chemical composition of the pottery shards from the Yiting Site in the Yongcheng area.

Statistical Measures	SiO_2_	Al_2_O_3_	Fe_2_O_3_	K_2_O	MgO	CaO	Na_2_O	TiO_2_
Number of Samples	24	24	24	24	24	24	24	24
Mean	67.29	17.36	5.98	3.49	1.72	1.41	1.25	0.81
Maximum	69.01	18.08	7.11	4.32	1.98	1.81	1.52	0.93
Minimum	65.19	15.88	5.28	3.11	1.41	0.84	0.96	0.70
Standard Deviation	1.30	0.62	0.59	0.34	0.19	0.28	0.16	0.06

## Data Availability

The original contributions presented in the study are included in the article; further inquiries can be directed to the corresponding author.
